# Reviewer acknowledgement 2012

**DOI:** 10.1186/1757-1146-6-4

**Published:** 2013-02-19

**Authors:** Hylton Menz, Mike Potter

**Affiliations:** 1 La Trobe University, Victoria, Australia; 2 University of Southampton, Southampton, UK

## Abstract

**Contributing reviewers:**

The Editors of *Journal of Foot and Ankle Research* would like to thank all our reviewers who have contributed to the journal in Volume 5 (2012).

## 

Rami Abboud

United Kingdom

Christian Barton

Australia

Shan Bergin

Australia

Daniel Bonanno

Australia

Catherine Bowen

United Kingdom

Chris Bower

United Kingdom

Helen Branthwaite

United Kingdom

Ivan Bristow

United Kingdom

Joshua Burns

Australia

Sicco Bus

Netherlands

Paul Butterworth

Australia

Chi Wai Cheung

Hong Kong

Bavornrit Chuckpaiwong

Thailand

Vivienne Chuter

Australia

Natalie Collins

Australia

Jill Dawson

United Kingdom

Beverley Durrant

United Kingdom

Angela Margaret Evans

New Zealand

Mark Gilheany

Australia

Ward Glasoe

United States of America

Ali Hajeer

Saudi Arabia

Edwin Harris

United States of America

Anna Hatton

Australia

Gordon Hendry

Australia

Rebecca Kearney

United Kingdom

Tim Kilmartin

Ireland

Peter Anthony Lazzarini

Australia

Magnus Löndahl

Sweden

Wendy Lowe

United Kingdom

Christopher Maclean

Canada

Nicola Maffulli

United Kingdom

Anthony John Maher

United Kingdom

Alistair Mcinnes

United Kingdom

Patrick Mckeon

United States of America

Jasmine Menant

Australia

Karen Mickle

Australia

Kevin Miller

United States of America

Marco Alessandro Minetto

Italy

Stewart Morrison

United Kingdom

Michael Mueller

United States of America

Shannon Munteanu

Australia

George S Murley

Australia

Susan Nancarrow

Australia

Aziz Nather

Singapore

Christopher Nester

United Kingdom

Christopher Neville

United States of America

Vanessa Nube

Australia

William Parr

Australia

Byron Perrin

Australia

Julia Potter

United Kingdom

Trevor D Prior

United Kingdom

Smita Rao

United States of America

Anita Raspovic

Australia

Michael Skovdal Rathleff

Denmark

Anthony Redmond

United Kingdom

Lloyd Reed

Australia

Max Reijman

Netherlands

Ana Paula Ribeiro

Brazil

Caroline Robinson

Australia

Keith Rome

New Zealand

Dieter Rosenbaum

Germany

Thomas Roukis

United States of America

Rolf Scharfbillig

Australia

Paul Scherer

United States of America

Deborah Schoen

Australia

Eric Senneville

France

Alison September

South Africa

Cliff Shearman

United Kingdom

Michelle Spruce

United Kingdom

Maria Stokes

United Kingdom

Kazuomi Sugamoto

Japan

Kristian Thorborg

Denmark

Stephen Urry

Australia

Karen Annette Vinall

United Kingdom

Tracey Vlahovic

United States of America

Scott Wearing

Australia

Meredith Wilkinson

Australia

Cylie Williams

Australia

Anita Williams

United Kingdom

